# Real-world efficacy and safety of cefepime for pediatric community-acquired pneumonia: a propensity score-matched study

**DOI:** 10.3389/fcimb.2025.1616184

**Published:** 2025-06-18

**Authors:** Changxin Liu, Jiayu Deng, Yudi Xing, Yanqing Song

**Affiliations:** ^1^ School of Pharmaceutical Sciences, Jilin University, Changchun, China; ^2^ Department of Pharmacy, Lequn Branch, The First Hospital of Jilin University, Changchun, China

**Keywords:** cefepime, pediatric community-acquired pneumonia, antibiotic efficacy, propensity score matching, microbiological clearance, adverse drug reactions, real-world evidence

## Abstract

**Objective:**

This study aimed to evaluate the real-world clinical efficacy and safety of cefepime in treating pediatric community-acquired pneumonia (CAP), comparing it with other broad-spectrum antibiotics, including cefoperazone-sulbactam and meropenem, using a propensity score-matched design.

**Methods:**

A retrospective, propensity score-matched cohort study was conducted in pediatric patients (0–18 years) hospitalized with CAP. Patients treated with cefepime were compared to those treated with cefoperazone-sulbactam or meropenem. Clinical outcomes, microbiological clearance, and adverse events were assessed, and propensity score matching was applied to minimize confounding.

**Results:**

A total of 788 patients were included, with 720 in the cefepime group and 68 in the comparator group. Both groups showed comparable clinical efficacy, with no significant differences in symptom resolution, laboratory normalization, or radiographic improvement. Microbiological clearance rates were also similar between the groups. The incidence of adverse events was low in both groups, and no statistically significant difference in adverse events was observed between cefepime and the comparator group.

**Conclusion:**

Our results suggest that cefepime is a clinically effective and well-tolerated alternative to other broad-spectrum antibiotics for pediatric CAP, demonstrating comparable clinical outcomes and safety profiles. These findings support cefepime as a viable empiric therapy option, particularly in settings with limited microbiological diagnostics. Further studies are needed to confirm these results and optimize dosing strategies for pediatric populations.

## 1 Introduction

Pneumonia is one of the major acute respiratory infections worldwide and is the most common single cause of death in children under five; this trend is particularly observed in low- and middle-income countries (Rudan et al., 2008; McAllister et al., 2019). According to the World Health Organization (WHO), pneumonia accounts for nearly 15% of all deaths in children under five years old globally (Rahman et al., 2021; Zhou et al., 2022). In CAP burden pediatric community-acquired China has been increasing steadily over several years, with great adverse effects on health care supply, necessitating promptly improving treatment plans (Zhang et al., 2013; Qian et al., 2024).

The 2019 Global Burden of Disease (GBD) study showed that pneumonia had a bimodal age distribution; the peaks were in children younger than five and adults older than seventy (Collaborators, 2017). This has also been seen in other large national studies within China. In urban areas, the incidence of CAP was reported as 7.13 per 1,000 person-years, with children being particularly at risk because their immune systems are not fully developed (Sun et al., 2020). These immunological deficits make pediatric patients susceptible to both typical and atypical pathogens, and this will further complicate diagnoses, increasing the need for prompt empirical treatment (Cao et al., 2023; Chen et al., 2024). As a result, pediatric CAP often advances rapidly, very frequently needing hospitalization and long-term care (Goodman et al., 2019).

Etiological studies conducted across mainland China have identified common causative bacterial pathogens in children with CAP, including *Streptococcus pneumoniae*, *Haemophilus influenzae*, *Staphylococcus aureus*, *Klebsiella pneumoniae*, *Escherichia coli*, and *Enterobacter cloacae* (Ning et al., 2017; Fu et al., 2021). International and Chinese clinical guidelines consistently recommend early initiation of empirical antimicrobial therapy, typically involving β-lactams and macrolides, to ensure adequate coverage of both Gram-positive and Gram-negative bacteria (Bradley and Arrieta, 2001; File and Niederman, 2004).

Of the β-lactams, cefepime—a fourth-generation cephalosporin—has shown good activity against many pathogens typically associated with pneumonia. It was approved in the early 1990s for the treatment of pneumonia and other serious infections and is now recommended by several guidelines for the management of hospital-acquired pneumonia (HAP) (Erb et al., 2016; Torres et al., 2017). While cefepime has been used extensively in the treatment of adult CAP, its clinical efficacy and safety profile in pediatric populations with CAP are not well documented, especially in real-world settings. This lack of information is worrisome, especially considering that rates of antimicrobial resistance are climbing coincidentally with broader-spectrum antibiotic use in this pediatric population.

Recent surveillance data suggest that inadequate antibiotic stewardship has led to a growing resistance among Gram-negative pathogens in children with pneumonia, further complicating empirical treatment decisions (Mai et al., 2023; Shi et al., 2023). Cefepime has been considered a potential option due to its robust antibacterial spectrum and favorable pharmacokinetic properties, including excellent tissue penetration and stability against most β-lactamases. However, concerns regarding its neurotoxicity, especially in vulnerable populations like children, call for cautious and evidence-based clinical application (Li et al., 2019).

To address these gaps, this study retrospectively evaluates the real-world efficacy, safety, and microbial clearance outcomes of cefepime in the treatment of pediatric CAP. Using a propensity score-matched (PSM) design, we compare cefepime with other broad-spectrum regimens—namely, cefoperazone-sulbactam and meropenem—in hospitalized children. These findings aim to inform clinical drug selection and pediatric antimicrobial stewardship, providing context-specific insights for managing CAP in high-resistance settings such as China.

## 2 Methods

### 2.1 Study design and data source

This single-center, retrospective, propensity score-matched (PSM) cohort study was conducted at The First Hospital of Jilin University, Lequn Branch, covering admissions from January 1, 2023, to December 31, 2023. Patient-level data were retrieved from the hospital’s Health Information System (HIS), including demographics (age, gender), clinical diagnoses, medication orders (drug name, dosage, frequency, duration), and laboratory results [white blood cell count (WBC), neutrophil percentage (NE%), procalcitonin (PCT), and C-reactive protein (CRP)] (Bradley et al., 2011; Florin et al., 2020).

All relevant data were extracted into a structured case report form (CRF) developed to ensure consistency in data collection and endpoint classification. Ethical approval was obtained from the Ethics Committee of The First Hospital of Jilin University (Approval No. 2024-1030). Given the retrospective nature of the study and anonymization of patient data, the requirement for informed consent was waived.

### 2.2 Patient selection and group assignment

Eligible patients were hospitalized children (aged 0–18 years) diagnosed with CAP, defined as a lower respiratory tract infection acquired outside healthcare settings. Inclusion criteria required receipt of cefepime or cefoperazone-sulbactam/meropenem for ≥72 hours. Patients were excluded if they: (1) lacked a confirmed diagnosis of CAP; (2) received antibiotics for <72 hours; (3) were treated with multiple antimicrobial agents simultaneously for >72 hours; or (4) had severe comorbidities (e.g., cardiac, neurological, or gastrointestinal disorders).

Severe comorbidities were defined as the presence of any of the following: (1) congenital heart disease requiring surgery or heart failure with reduced ejection fraction (EF < 40%) or NYHA class III–IV; (2) epilepsy requiring long-term therapy or ≥2 seizures within 6 months, or cerebral palsy with significant functional limitation; (3) active inflammatory bowel disease requiring immunosuppressive treatment; (4) primary immunodeficiency or chronic immunosuppressive therapy (e.g., corticosteroids ≥20 mg/day for ≥4 weeks). These were determined through comprehensive review of electronic medical records, including diagnostic codes, laboratory values, imaging findings, and medication records.

**Figure 1:**
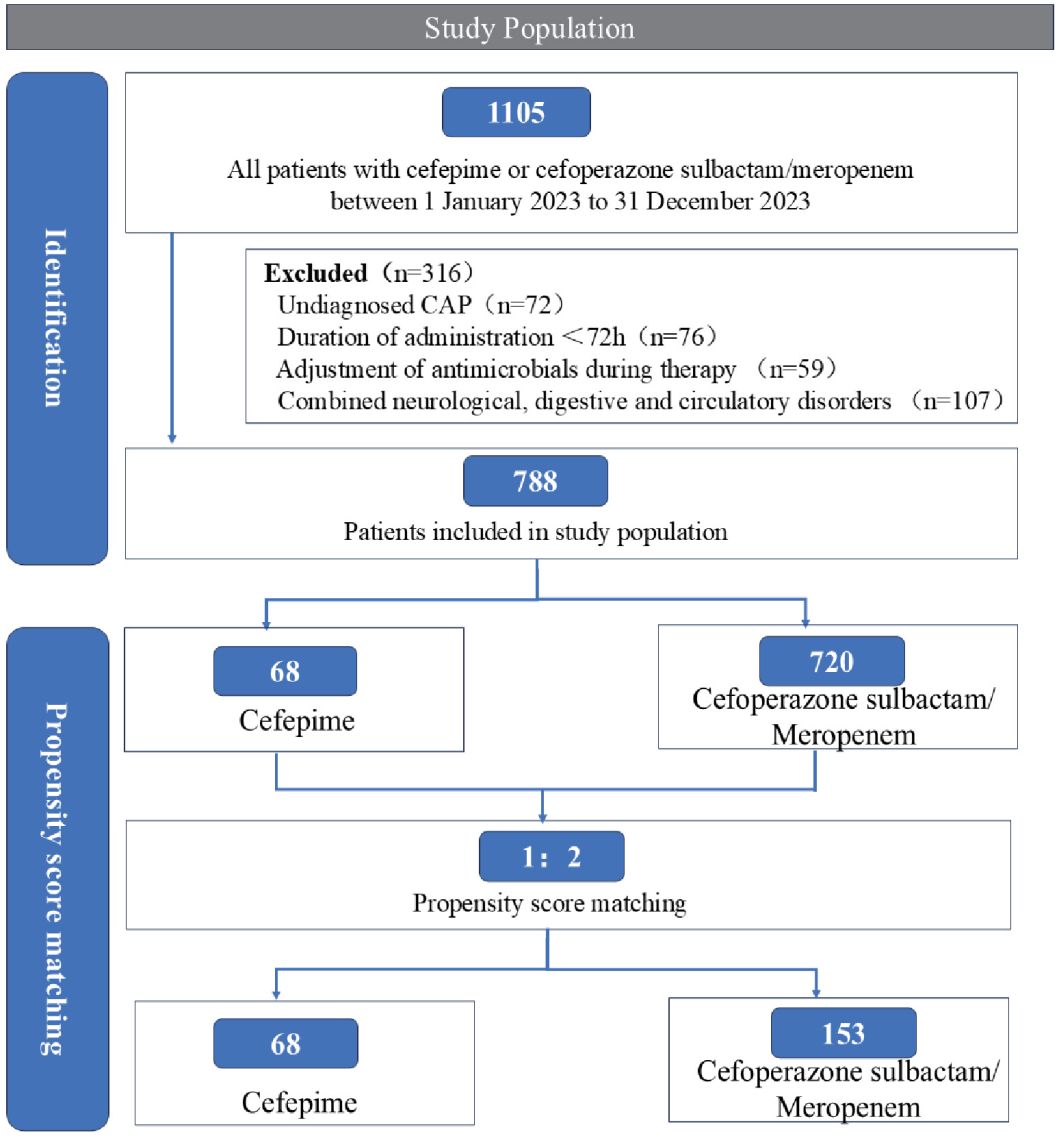
Flowchart illustrating the selection process of the study population.

A detailed patient selection process is illustrated in **Figure 1**.

Patients receiving cefepime constituted the intervention group, while those treated with cefoperazone-sulbactam or meropenem formed the comparator group. To reduce confounding bias and enhance comparability between groups, PSM was performed based on baseline characteristics (Liang et al., 2021).

### 2.3 Outcome definitions

The primary and secondary outcomes of the study included clinical efficacy, microbiological clearance, and the incidence of adverse drug reactions (ADRs). Clinical efficacy was assessed in accordance with the Guidance for Clinical Trials of Anti-bacterial Drugs (Drugs, 2014). Patients were classified as “cured” if they demonstrated complete resolution of clinical signs and symptoms—such as fever, cough, and respiratory distress—alongside normalization of infection-related laboratory parameters, including white blood cell count (WBC), neutrophil percentage (NE%), procalcitonin (PCT), and C-reactive protein (CRP). Radiological improvement based on chest imaging was also required for this classification. An “improvement” classification was assigned to patients who exhibited partial clinical and laboratory recovery but had persistent abnormalities in one or more parameters. Cure and improvement status were assessed based on the patient’s full course of hospitalization and not limited to a fixed timepoint. Cases were deemed “ineffective” if there was no significant clinical improvement or if the patient’s condition deteriorated after 72 hours of antibiotic therapy.

Microbiological clearance was evaluated through culture results of respiratory and blood specimens obtained before and after treatment. Clearance was defined as the eradication of the causative pathogen from the site of infection as evidenced by negative culture findings. Microbiological clearance was defined by a single negative post-treatment culture where available, in line with typical clinical documentation practices. In the absence of follow-up cultures, presumed clearance was inferred from documented clinical resolution. Repeat cultures were not systematically obtained unless clinically indicated, consistent with routine pediatric management. Presumed clearance was applied in cases where clinical cure was achieved but post-treatment culture was unavailable, either due to resolution of symptoms or the infeasibility of invasive sampling. Non-clearance referred to the persistence of the original pathogen in follow-up cultures, while presumed non-clearance denoted clinical failure in the absence of repeat microbiological testing.

The evaluation of ADRs was independently conducted by two investigators who reviewed medical records for descriptions of potential drug-related events using predefined criteria. The causality of each ADR was assessed and classified as “certain,” “probable,” or “possible” based on established pharmacovigilance frameworks, and only those events falling within these categories were included in the safety analysis. The severity of ADRs was graded according to the Common Terminology Criteria for Adverse Events (CTCAE), version 5.0 (Varona et al., 2023). Discrepancies in ADR assessment between the two reviewers were resolved through discussion to reach consensus. While blinded adjudication was not feasible due to the retrospective nature of the study, the use of independent dual-review and predefined attribution criteria helped minimize potential bias and enhance the objectivity of causality assessment.

### 2.4 Statistical analysis

Data were pre-processed using Microsoft Excel and analyzed with IBM SPSS 26.0. Continuous variables were expressed as mean ± standard deviation (SD) or median with interquartile range (IQR) as appropriate. Normality of continuous variables was assessed using the Shapiro–Wilk test. Independent-sample t-tests were used when data were normally distributed; otherwise, Wilcoxon rank-sum tests were applied. Between-group comparisons were made using independent-sample t-tests or Wilcoxon rank-sum tests. Categorical variables were compared using Chi-square or Fisher’s exact tests. A two-sided P value < 0.05 was considered statistically significant.

To control for potential confounding, PSM was performed using nearest-neighbor matching with a caliper width of 0.2 and a 1:2 matching ratio (intervention: control). Matching was based on demographic and baseline clinical variables (age, gender, symptom profile, laboratory results, and imaging findings). Covariate balance post-matching was assessed via standardized mean differences (SMDs) and multivariate imbalance measure L1 (Iacus et al., 2009, 2012). Due to the limited size of the comparator group (n = 68), a 1:2 nearest-neighbor matching with a caliper width of 0.2 was applied to optimize covariate balance while retaining statistical power. Some cefepime-treated patients could not be matched due to substantial baseline differences in age, symptom severity, laboratory abnormalities, and radiographic findings. As a result, only 135 cefepime patients were retained in the matched cohort. Despite this reduction, post-matching diagnostics (e.g., SMD < 0.1 and L1 statistics) demonstrated strong covariate balance, and key outcome measures remained consistent between groups. Although the reduced matched sample may limit the power for subgroup analyses, the primary objective of evaluating the non-inferiority of cefepime was preserved.

Missing data were handled using complete case analysis. Patients with incomplete baseline variables necessary for matching or outcome determination were excluded from the final analytic dataset. No imputation methods were used.

While unmatched cohort data were presented to demonstrate baseline imbalances and offer transparency, all outcome comparisons and conclusions were derived from the PSM population.

To account for multiple comparisons across five key secondary outcomes, clinical response, laboratory normalization, radiographic improvement, microbiological clearance, and adverse events, a Bonferroni correction was applied. The corrected significance threshold was set at *P* < 0.01 to control the family-wise error rate.

## 3 Results

### 3.1 Baseline characteristics

Out of 788 pediatric patients diagnosed with CAP, a total of 135 cefepime-treated patients and 68 patients treated with either cefoperazone-sulbactam or meropenem were successfully matched using a 1:2 PSM approach. Matching variables included age, gender, symptom profile, laboratory indicators, and radiographic findings.

After matching, baseline characteristics were well balanced between groups. The mean age was 6.09 ± 3.06 years in the cefepime group and 6.44 ± 3.16 years in the comparator group, with no significant difference (*P* = 0.582). Age was used as a continuous covariate in the matching process to avoid information loss and residual confounding, while categorical age bands are presented in **Table 1** for clinical clarity. Gender distribution (male: 54.1% vs. 52.9%, *P* = 0.879), treatment duration (median: 6 days in both groups, *P* = 0.764), laboratory abnormalities (*P* = 0.808), and radiographic findings (P = 0.727) were also similar between groups. Detailed baseline comparisons are shown in **Table 1**; **Supplementary Tables S1**, **S2**, and **Supplementary Figures S1**, **S2**.

**Table 1:** Baseline characteristics of pediatric CAP patients before and after propensity score matching (N = 788).

Variables	Before PSM		*P*	After PSM		*P*	Effect Size (Mean Diff or OR, 95% *CI*)
	Cefepime Group (N = 720)	Comparator Group (N = 68)		Cefepime Group (N = 135)	Comparator Group (N = 68)		
Age, years (mean ± SD)	6.20 ± 3.15	6.44 ± 3.16	0.538	6.09 ± 3.06	6.44 ± 3.16	0.582	Mean diff: -0.24 (-1.03-0.55)
Male, n (%)	367 (51.0)	36 (52.9)	0.756	73 (54.1)	36 (52.9)	0.879	OR: 0.92 (0.56-1.52)
Treatment duration, median (IQR)	7 (5, 9)	6 (5, 8)	0.047	6 (4, 8)	6 (5, 8)	0.764	Median diff ≈ 0 (ns)
Symptoms present, n (%)	719 (99.9)	68 (100.0)	1.000	135 (100.0)	68 (100.0)	1.000	Not estimable (100% in both groups)
Laboratory abnormalities, n (%)	311 (43.2)	44 (64.7)	<0.001	85 (63.0)	44 (64.7)	0.808	OR: 0.41 (0.25–0.70)
Radiographic findings, n (%)	414 (57.5)	34 (50.0)	0.233	71 (52.6)	34 (50.0)	0.727	OR: 1.24 (0.72–2.14)

Comparator group includes patients treated with cefoperazone-sulbactam or meropenem. PSM was conducted using nearest-neighbor matching (1:2 ratio, caliper = 0.2). Laboratory and imaging data coded as binary variables: 0 = normal/no abnormality, 1 = abnormal. *P* values based on the Chi-square test for categorical variables and the Wilcoxon rank-sum test for medians.

### 3.2 Clinical efficacy

Both groups demonstrated complete clinical response (100% in both cefepime and comparator groups), with no statistically significant differences observed across symptom resolution, laboratory normalization, or radiologic improvement (**Table 2**). Among the matched cohort, all patients achieved symptomatic relief within the treatment window. Normalization of infection-related biomarkers (WBC, NE%, PCT, CRP) was observed in 95.6% of patients in the cefepime group and 100% in the comparator group (*P* = 0.375). Radiographic improvement was noted in 100% of patients treated with cefepime, compared to 97.1% in the comparator group (*P* = 0.324). Overall, clinical outcomes indicated that cefepime was non-inferior to cefoperazone-sulbactam or meropenem in this pediatric CAP cohort.

**Table 2:** Comparison of clinical efficacy outcomes between cefepime and comparator groups before and after propensity score matching.

Outcome	Before PSM		*P*	After PSM		*P*
	Cefepime Group (N = 720)	Comparator Group (N = 68)		Cefepime Group (N = 135)	Comparator Group (N = 68)	
Overall Clinical Response, n/N (%, 95% CI)	720/720 (100.0, 99.5%-100.0%)	68/68 (100.0, 94.7%-100.0%)	–	135/135 (100.0, 97.3%-100.0%)	68/68 (100.0, 94.7%-100.0%)	–
Symptom Relief, n/N (%, 95% CI)	718/720 (99.7, 99.0%-100.0%)	8/68 (100.0, 94.7%-100.0%)	–	135/135 (100.0, 97.3%-100.0%)	68/68 (100.0, 94.7%-100.0%)	–
Cure, n/N (%)	29/720 (4.0)	6/68 (8.8)		0/135 (0.0)	6/68 (8.8)	
Improvement, n/N (%)	689/720 (95.7)	62 (91.2)		135/135 (100.0)	62 (91.2)	
Ineffective, n/N (%)	2/720 (0.3)	0/68 (0.0)		0/135 (0.0)	0/68 (0.0)	
Laboratory Normalization, n/N (%, 95% CI)	301/311 (96.8, 94.2%-98.4%)	44/44 (100.0, 92.0%-100.0%)	0.682	108/113 (95.6, 90.0%-98.5%)	44/44 (100.0, 92.0%-100.0%)	0.375
Cure, n/N (%)	6/311 (1.9)	0/44 (0.0)		2/113 (1.8)	0/44 (0.0)	
Improvement, n/N (%)	295/311 (94.9)	44/44 (100.0)		106 (93.8)	44/44 (100.0)	
Ineffective, n/N (%)	10/311 (3.2)	0/44 (0.0)		5/113 (4.4)	0/44 (0.0)	
Radiologic Improvement, n/N (%, 95% CI)	414/414 (100.0, 99.1%-100%)	33/34 (97.1, 84.7%-99.9%)	0.076	71/71 (100.0, 94.9%-100.0%)	33/34 (97.1, 84.7%-99.9%)	0.324
Improvement, n/N (%)	414/414 (100.0)	33/34 (97.1)		71/71 (100.0)	33/34 (97.1)	
Ineffective, n/N (%)	0/414 (0.0)	1/34 (2.9)		0/71 (0.0)	1/34 (2.9)	

Clinical cure was defined as complete resolution of signs and symptoms, normalization of laboratory biomarkers (WBC, NE%, PCT, CRP), and radiographic improvement. Improvement indicated partial recovery, and ineffective indicated lack of meaningful change after 72 hours of therapy. Comparator group includes patients treated with cefoperazone-sulbactam or meropenem. A Bonferroni-corrected significance threshold of *P* < 0.01 was applied for multiple comparisons across five secondary outcomes.

To further explore potential treatment heterogeneity, we conducted an exploratory subgroup analysis stratified by baseline age and by individual comparator regimens (cefoperazone–sulbactam, meropenem, and dual-use). No statistically significant differences were found in clinical cure rates, laboratory normalization, or radiologic improvement across subgroups. These results support the consistency of cefepime’s clinical effectiveness across pediatric age groups and comparator antibiotic types. Full results are provided in the Supporting Information (**Supplementary Tables S3**, **S4**).

### 3.3 Microbiological findings

Microbiological sampling was performed on 47.72% of the study cohort (376/788). Among the blood cultures (362/788), the positivity rate was low, with only 3 positive results (0.83%) yielding isolates of *Staphylococcus aureus* and *Staphylococcus hominis*. In these cases, all cefepime-treated patients who had positive blood cultures were considered to have achieved presumed microbiological clearance based on clinical recovery. Sputum cultures were conducted in 14 patients (1.78%), of which 6 yielded pathogenic organisms, including *Enterobacter cloacae*/*Pseudomonas aeruginosa, Staphylococcus aureus, Pseudomonas malodorous*, and *Haemophilus influenzae*. Among the cefepime group, four patients achieved presumed clearance, while one patient showed presumed non-clearance. The control group had one case of confirmed microbiological eradication. Detailed microbiological findings are summarized in **Table 3**.

**Table 3:** Microbiological clearance and pathogen eradication outcomes in pediatric CAP patients.

Microbiological Examination	Detection rate	Microbiological	Microbiological Clearance
Microbiology Culture Rate, n/N (%)	376/788 (47.7)		
Blood Culture Rate, n/N (%)	362/788 (45.9)		
Negative Results Rate, n/N (%)	359/362 (99.2)		
Positive Results	Cefepime Group	*Staphylococcus hominis*	Assumed Clearance
		*Staphylococcus hominis*	Assumed Clearance
		*Staphylococcus aureus*	Assumed Clearance
Sputum Culture Rate, n/N (%)	14/788 (1.78)		
Negative Results Rate, n/N (%)	8/14 (57.14)		
Positive Results	Cefepime Group	*Staphylococcus aureus*	Assumed Clearance
		*Staphylococcus aureus*	Assumed Clearance
		*Pseudomonas aeruginosa*	Assumed Non-clearance
		*Enterobacter cloacae*	Assumed Clearance
		*Hemophilus influenzae*	Assumed Clearance
	Comparator Group	*Enterobacter cloacae*/*Pseudomonas aeruginosa*	Clearance

Microbiological data are descriptive only; due to the limited number of positive culture cases, no statistical comparisons were made. All cultures were collected prior to or at the initiation of antibiotic treatment. Timing of follow-up sampling was inconsistently documented and not analyzed.

### 3.4 Adverse events and safety evaluation

A total of 57 adverse events (AEs) were reported, of which 46 (80.7%) were considered to be related to antibiotic treatment. In the Cefepime Group, 5.7% of patients (41/720) experienced ADRs, compared to 11.8% (8/68) in the Comparator Group (*P* = 0.062). Most events were mild to moderate in severity, with 48.8% classified as Grade 1 and 51.2% as Grade 2 in the Cefepime Group. Reported ADRs primarily involved dermatologic, gastrointestinal, and hepatobiliary systems. No Grade ≥3 ADRs were reported in either group. The difference in ADR severity distributions between groups was not statistically significant (*P* = 0.467). A summary of adverse event types, severity grades, and affected organ systems is provided in **Table 4**.

**Table 4:** Adverse drug reactions and safety outcomes in pediatric CAP patients before and after propensity score matching.

Adverse Event	Before PSM		*P*	After PSM		*P*
	Cefepime-related (N = 720)	Cefoperazone-sulbactam/Meropenem-related (N = 68)		Cefepime-related (N = 135)	Cefoperazone-sulbactam/Meropenem-related (N = 68)	
Number of ADR^+^, n/N (%, 95% CI)	41/720 (5.7, 4.1%-7.6%)	8/68 (11.8, 5.2%-21.9%)	0.062	7/135 (5.2, 2.1%-10.4%)	8/68 (11.8, 5.2%-21.9%)	0.152
Degree of Association^*^, n/N (%)						
Certain	6/41 (14.6)	3/8 (37.5)		5/7 (71.4)	3/8 (37.5)	
Probable	/	/		/	/	
Possible	35/41 (85.4)	5/8 (62.5)		2/7 (28.6)	5/8 (62.5)	
Level of severity^*^, n/N (%)			0.0073			0.467
Grade 1	20/41 (48.8)	8/8 (100.0)		6/7 (85.7)	8/8 (100.0)	
Grade 2	21/41 (51.2)	0/8 (0.0)		1/7 (14.3)	0/8 (0.0)	
Involved systems^*^, n/N (%)						
Heart	5/41 (12.2)	2/8 (25.0)		/	2/8 (25.0)	
Skin	11/41 (26.8)	6/8 (75.0)		6/7 (85.7)	6/8 (75.0)	
Gastrointestinal	17/41 (41.5)	/		/	/	
Hepatobiliary	4/41 (9.8)	/		/	/	
Systemic and General	2/41 (4.9)	/		1/7 (14.3)	/	
Blood system	2/41 (4.9)	/		/	/	

ADR^+^ refers to adverse drug reactions considered definitely, probably, or possibly related to the drug. The degree of association was classified as certain, probable, or possible based on clinical assessment. ADR severity was graded according to the Common Terminology Criteria for Adverse Events (CTCAE), version 5.0. System involvement was categorized by the primary organ system affected, as documented in the medical record. P-values were calculated using Fisher’s exact test for all categorical comparisons due to small subgroup sizes. Confidence intervals for proportions were computed using the Clopper–Pearson (exact binomial) method.

After propensity score matching, the incidence of ADRs was 5.2% (7/135) in the cefepime group and 11.8% (8/68) in the comparator group (*P* = 0.152, Fisher’s exact test). The absolute risk difference was -6.6% (95% CI: -15.1% to 1.9%), suggesting a lower ADR rate with cefepime, although the difference was not statistically significant.

## 4 Discussion

This retrospective cohort study provides real-world evidence that cefepime demonstrates comparable clinical efficacy and safety to other commonly used broad-spectrum antibiotics, namely cefoperazone-sulbactam and meropenem, in the treatment of pediatric CAP. These findings support the potential role of cefepime as an appropriate empiric option in pediatric CAP management, particularly in high-burden regions such as China where timely, effective antibiotic selection is critical.

Children possess immunologically immature systems that limit effective pathogen clearance, predisposing them to more rapid disease progression and a higher risk of complications in respiratory infections (Patria and Esposito, 2013). Cefepime, a fourth-generation cephalosporin, offers a broad spectrum of activity and pharmacokinetic stability, including reliable penetration into lung tissue and efficacy against AmpC β-lactamase-producing Enterobacteriaceae (Herrmann et al., 2024). In the present study, comparable clinical outcomes—including resolution of symptoms, normalization of inflammatory markers, and radiographic improvement—were observed between treatment groups, suggesting that cefepime is clinically effective in pediatric populations.

In the context of escalating global antimicrobial resistance, the judicious selection of empiric antibiotics is essential to stewardship frameworks. The comparable efficacy observed in this study suggests that cefepime may serve as a viable alternative to carbapenems in the management of non-severe pediatric CAP, thereby preserving last-line agents for confirmed or highly suspected multidrug-resistant infections. This approach may help delay the emergence of resistance while maintaining clinical efficacy. However, optimal empiric therapy should be guided by local resistance patterns, disease severity, and patient-specific risk factors. Integrating cefepime into empiric treatment protocols for selected pediatric patients may also reduce the need for combination regimens, minimizing drug burden and associated toxicities. Although the incidence of ADRs was numerically lower in the cefepime group following propensity score matching, the difference did not reach statistical significance. The absolute risk difference was -6.6% (95% CI: -15.1% to 1.9%), indicating that cefepime may have a comparable or potentially more favorable safety profile than the comparator agents. However, given the limited sample size and wide confidence interval, this finding should be interpreted with caution and confirmed in larger prospective studies.

The predominant bacterial pathogens associated with pediatric CAP in China include *Streptococcus pneumoniae*, *Haemophilus influenzae*, and various Gram-negative organisms. In many primary care and secondary hospital settings, access to microbiological diagnostics remains limited, often delaying definitive pathogen identification. The findings of this study provide clinically relevant insights for such settings, where cefepime’s broad coverage may reduce reliance on empiric dual-agent therapy. Moreover, the findings of this study are consistent with the 2019 Chinese Guidelines for the Diagnosis and Treatment of Pediatric Community-Acquired Pneumonia, which emphasize the importance of early empiric antimicrobial therapy tailored to likely pathogens based on age, clinical severity, and epidemiological context.

Cefepime-associated neurotoxicity, including encephalopathy, seizures, and altered mental status, has been increasingly reported in vulnerable populations, particularly in patients with impaired renal function due to reduced drug clearance. While such events are more commonly documented in adults, emerging data suggest pediatric patients, especially those with compromised renal function or concomitant nephrotoxic therapies, may also be at risk. Therefore, careful dosing and renal function monitoring are essential when administering cefepime in these subgroups (Taylor et al., 2025; Tseng et al., 2025; Yamaguchi et al., 2025).

Emerging spatial multi-omics technologies offer promising avenues for advancing our understanding of pediatric infectious diseases. For instance, spatial-CITE-seq enables high-plex protein and whole transcriptome co-mapping at cellular resolution, facilitating detailed analysis of tissue-specific immune responses (Liu et al., 2023). Multimodal tri-omics mapping has been employed to elucidate the spatial dynamics of mammalian brain development and neuroinflammation, demonstrating the potential of integrating multiple omics layers to study complex biological processes (Zhang et al., 2024). Additionally, Perturb-DBiT allows for spatially resolved *in vivo* CRISPR screen sequencing, providing insights into gene function within the native tissue context (Baysoy et al., 2024). Incorporating these advanced methodologies in future studies could significantly enhance our understanding of pathogen-host interactions and inform the development of targeted therapeutic strategies.

## 5 Limitations and future directions

This study has several limitations. First, the single-center retrospective design may restrict the generalizability of findings beyond the study setting. Although PSM was employed to balance baseline covariates, unmeasured confounding variables, such as pathogen characteristics, comorbidities, and concomitant therapies, may still have influenced outcomes. Additionally, we did not perform sensitivity analyses (e.g., caliper width variation or E-value estimation), which limits the ability to assess the robustness of causal inferences. Future studies with larger, multicenter datasets should integrate such methods to enhance analytical rigor.

Second, while PSM achieved good balance between groups, the reduction in sample size, particularly in the cefepime group, may have limited statistical power for subgroup analyses. Similarly, the exclusion of patients who received multiple antibiotics for over 72 hours, although necessary to isolate treatment effects, may have introduced selection bias by excluding more severe or treatment-refractory CAP cases. These constraints should be addressed in future prospective cohort designs.

Third, the analysis of microbiological outcomes was limited by low positivity rates and incomplete culture data. Specimen collection in pediatric populations is often challenging due to low sputum yield and prior antibiotic exposure, which may reduce culture sensitivity. Moreover, the precise timing of culture collection relative to treatment initiation and resolution was inconsistently recorded. As such, no inferential statistical comparisons were made for microbiological clearance due to the very small number of confirmed infections. These limitations were noted in **Table 3** and discussed to prevent overinterpretation. Future studies should incorporate molecular diagnostic tools and standardized timing of specimen collection to improve microbiological evaluation.

Fourth, another limitation of this study relates to the heterogeneity within the comparator group, which included both cefoperazone–sulbactam and meropenem. Although these agents are both recommended by national pediatric CAP guidelines as empirical options for patients with severe or resistant infections, and their use often overlaps in clinical practice, they represent different antibiotic classes with potentially distinct efficacy and safety profiles. To address this, we conducted an exploratory subgroup analysis comparing cefepime with cefoperazone–sulbactam, meropenem, and a small subset of patients who received both agents sequentially. While no statistically significant differences in clinical cure, laboratory normalization, or radiologic improvement were found, the sample size of the meropenem group (n = 19) and dual-use group (n = 2) was small, which may limit statistical power and the ability to detect subtle differences. Therefore, while our findings suggest overall comparability between cefepime and other broad-spectrum β-lactams, larger studies are warranted to confirm drug-specific outcomes with greater precision.

Fifth, while the overall incidence of ADRs was low, we reported confidence intervals for all ADR proportions to reflect statistical uncertainty due to small sample sizes. A Bonferroni correction was applied to control the family-wise error rate across multiple comparisons; however, findings from this exploratory study should still be interpreted cautiously. Additionally, causality assessments were based on retrospective chart review without blinded adjudication. To mitigate subjectivity, we used predefined pharmacovigilance criteria and independent dual-review, but prospective designs with blinded assessment would further strengthen safety evaluations.

Finally, the retrospective dataset precluded reliable time-to-event analyses, such as Kaplan–Meier estimation of symptom resolution or fever clearance, due to inconsistent documentation of symptom onset and resolution times. Future prospective studies should include structured symptom tracking to enable these analyses. Moreover, antibiotic susceptibility testing (AST) data were not consistently available in clinical records and thus were not included in our analysis. While this reflects real-world practice in pediatric care settings, it limits interpretation of microbiological efficacy in the context of resistance.

In future research, efforts should be made to validate these findings through prospective, multicenter studies that include broader pathogen detection methods, pharmacokinetic analyses of cefepime in pediatric populations, and further investigation of its neurotoxicity risk, particularly in children with renal impairment or those receiving nephrotoxic agents.

## 6 Conclusion

This research indicates the potential of cefepime as a treatment choice for children with CAP. Regarding the main outcome, the clinical efficacy and safety of cefepime and cefoperazone sulbactam/meropenem showed no notable variance in treating children with CAP. This work confirms its non-inferior efficacy and safety compared to broad-spectrum alternatives. Within antimicrobial stewardship frameworks, cefepime may serve as a first-line empiric choice for pediatric CAP in China, though its use must be guided by individualized risk assessment and dynamic pathogen surveillance. Future research should prioritize therapeutic optimization to balance efficacy, safety, and resistance mitigation.

## Data Availability

The raw data supporting the conclusions of this article will be made available by the authors, without undue reservation.
