# Supplementary material for: Real-world efficacy and safety of cefepime for pediatric community-acquired pneumonia: a propensity score-matched study
Source: Front Cell Infect Microbiol. 2025 Jun 18;15:1616184. doi: 10.3389/fcimb.2025.1616184 (PMC12213673; doi:10.3389/fcimb.2025.1616184)
Supplement: Supplementary file 1 [file DataSheet1.docx]

Table S1: Propensity Matching Score Sample Matching Results

| **Sample Sizes** | | | | | | | | |
| --- | --- | --- | --- | --- | --- | --- | --- | --- |
| Subsamples | All | | Matched | | Unmatched | | Discarded | |
|  | Treated | Control | Treated | Control | Treated | Control | Treated | Control |
| (all cases) | 720 | 68 | 135 | 68 | 585 | 0 | 0 | 0 |

Table S2: Relative multivariate imbalance before and after matching

| **Relative multivariate imbalance L1 (Iacus, King, & Porro, 2010)** | | |
| --- | --- | --- |
|  | Before matching | After matching |
| (all cases) | 0.710866013 | 0.639705882 |

Explanation:

The L1 measure statistic theoretically lies between 0 and 1. The smaller the L1 measure statistic before and after matching, the better the matching. In this study, the L1 measure statistic after matching was 0.6397 which was much smaller than the 0.7108 before matching, suggesting a good match


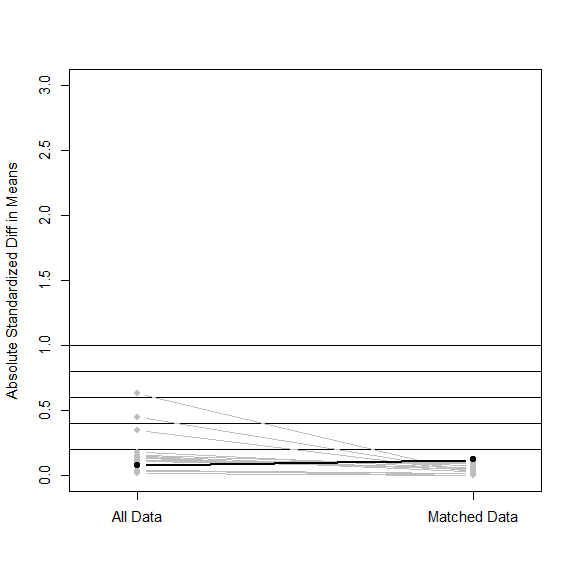


Figure S1: Line plot of individual differences

Explanation:

This figure shows the changes in the standardised differences of each covariate before and after matching. The line plot of individual differences shows that the standardised difference of each covariate is significantly reduced after matching, suggesting that the variables have reached balance and that the matching effect is good.


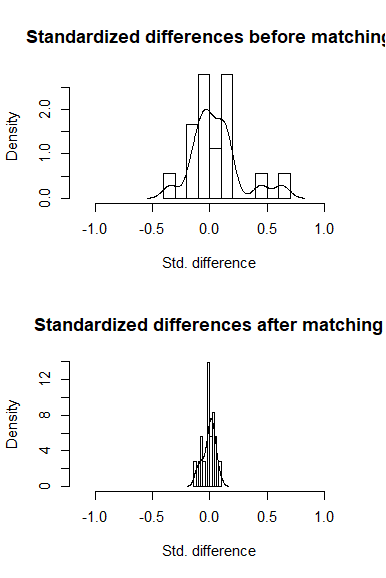


Figure S2: Histogram of standardized difference distribution

Explanation:

The standard deviation after matching is concentrated around 0, which can suggest that the matching has achieved a better result.

Table S3. Subgroup Analysis of Treatment Outcomes After Propensity Score Matching

| Outcomes | Cefepime (N = 135) | Cefoperazone-sulbactam (N = 47) | Meropenem (N = 19) | Dual-use (N = 2) | *P*  Cefepime vs. C-S | *P*  Cefepime vs. Meropenem | *P*  Cefepime vs. D-U |
| --- | --- | --- | --- | --- | --- | --- | --- |
| Overall Clinical Response, n/N (%) | 135/135 (100.0) | 47/47 (100.0) | 19/19 (100.0) | 2/2 (100.0) | 1.000 | 1.000 | 1.000 |
| Symptom Relief, n/N (%) | 135/135 (100.0) | 47/47 (100.0) | 19/19 (100.0) | 2/2 (100.0) | 1.000 | 1.000 | 1.000 |
| Cure, n/N (%) | 0/135 (0.0) | 2/47 (4.3) | 3/19 (15.8) | 1/2 (50.0) |  |  |  |
| Improvement, n/N (%) | 135/135 (100.0) | 45/47 (95.7) | 16/19 (84.2) | 1/2 (50.0) |  |  |  |
| Ineffective, n/N (%) | 0/135 (0.0) | 0/47 (0.0) | 0/19 (0.0) | 0/2 (0.0) |  |  |  |
| Laboratory Normalization, n/N (%) | 108/113 (95.6) | 28/28 (100.0) | 15/15 (100.0) | 1/1 (100.0) | 0.583 | 1.000 | 1.000 |
| Cure, n/N (%) | 2/113 (1.8) | 0/28 (0.0) | 0/15 (0.0) | 0/1 (0.0) |  |  |  |
| Improvement, n/N (%) | 106 (93.8) | 28/28 (100.0) | 15/15 (100.0) | 1/1 (100.0) |  |  |  |
| Ineffective, n/N (%) | 5/113 (4.4) | 0/28 (0.0) | 0/15 (0.0) | 0/1 (0.0) |  |  |  |
| Radiologic Improvement, n/N (%) | 71/71 (100.0) | 25/26 (96.2) | 7/7 (100.0) | 1/1 (100.0) | 0.268 | 1.000 | 1.000 |
| Improvement, n/N (%) | 71/71 (100.0) | 25/26 (96.2) | 7/7 (100.0) | 1/1 (100.0) |  |  |  |
| Ineffective, n/N (%) | 0/71 (0.0) | 1/26 (3.8) | 0/7 (0.0) | 0/1 (0.0) |  |  |  |

Dual-use refers to patients who were initially treated with cefoperazone–sulbactam and subsequently switched to meropenem.

C-S: Cefoperazone-sulbactam

D-U: Dual-use

P-values calculated using Fisher’s exact test.

Table S4. Subgroup Analysis of Clinical Efficacy Outcomes Between Cefepime and Comparator Groups After Propensity Score Matching, Stratified by Baseline Age

| Outcomes | Age | Cefepime  (N = 135) | Cefoperazone-sulbactam  (N = 47) | Meropenem  (N = 19) | Dual-use  (N = 2) |
| --- | --- | --- | --- | --- | --- |
| Overall Clinical Response, n/N (%) | ＜3 years | 23/135 (17.0) | 8/47 (17.0) | 2/19 (10.5) | / |
|  | 3-6 years | 53/135 (39.3) | 18/47 (38.3) | 7/19 (36.8) | / |
|  | 7-10 years | 47/135 (34.8) | 18/47 (38.3) | 10/19 (52.6) | / |
|  | 11-15 years | 12/135 (8.9) | 3/47 (6.4) | / | 2/2 (100.0) |
| Symptom Relief, n/N (%) |  | 135/135 (100.0) | 47/47 (100.0) | 19/19 (100.0) | 2/2 (100.0) |
|  | ＜3 years | 23/135 (17.0) | 9/47 (19.1) | / | / |
|  | Cure | / | 1/9 (11.1) | / | / |
|  | Improvement | 23/23 (100.0) | 8/9 (88.9) | / | / |
|  | Ineffective | / | / | / | / |
|  | 3-6 years | 53/135 (39.3) | 17/47 (36.2) | 8/19 (42.1) |  |
|  | Cure | / | / | 3/8 (37.5) | / |
|  | Improvement | 53/53 (100.0) | 17/17 (100.0) | 5/8 (62.5) | / |
|  | Ineffective | / | / | / | / |
|  | 7-10 years | 47/135 (34.8) | 17/47 (36.2) | 11/19 (57.9) | / |
|  | Cure | / | 1/17 (5.9) | / | / |
|  | Improvement | 47/47 (100.0) | 16/17 (94.1) | 11/11 (100.0) | / |
|  | Ineffective | / | / | / | / |
|  | 11-15 years | 12/135 (8.9) | 4/47 (8.5) | / | 2/2 (100.0) |
|  | Cure | / | / | / | 1/2 (50.0) |
|  | Improvement | 12/12 (100.0) | 4.4 (100.0) | / | 1/2 (50.0) |
|  | Ineffective | / | / | / | / |
| Laboratory Normalization, n/N (%) |  | 108/113 (95.6) | 28/28 (100.0) | 15/15 (100.0) | 1/1 (100.0) |
|  | ＜3 years | 18/113 (15.9) | 2/28 (7.1) | 1/15 (6.7) | / |
|  | Cure | / | / | / | / |
|  | Improvement | 18/18 (100.0) | 2/2 (100.0) | 1/1 (100.0) | / |
|  | Ineffective | / | / | / | / |
|  | 3-6 years | 49/113 (43.3) | 13/28 (46.4) | 4/15 (26.7) | / |
|  | Cure | 2/49 (4.1) | / | / | / |
|  | Improvement | 45/49 (91.8) | 13/13 (100.0) | 4/4 (100.0) | / |
|  | Ineffective | 2/49 (4.1) | / | / | / |
|  | 7-10 years | 37/113 (32.7) | 10/28 (35.7) | 10/15 (66.7) | / |
|  | Cure | / | / | / | / |
|  | Improvement | 35/37 (94.6) | 10/10 (100.0) | 10/10 (100.0) | / |
|  | Ineffective | 2/37 (5.4) | / | / | / |
|  | 11-15 years | 9/113 (8.0) | 3/28 (10.7) | / | 1/1 (100.0) |
|  | Cure | / | / | / | / |
|  | Improvement | 8/9 (88.9) | 3/3 (100.0) | / | 1/1 (100.0) |
|  | Ineffective | 1/9 (11.1) | / | / | / |
| Radiologic Improvement, n/N (%) |  | 71/71 (100.0) | 25/26 (96.2) | 7/7 (100.0) | 1 (100.0) |
|  | ＜3 years | 9/71 (12.7) | 3/26 (11.5) | 1/7 (14.3) | / |
|  | Improvement | 9/9 (100.0) | 3/3 (100.0) | 1/1 (100.0) | / |
|  | Ineffective | / | / | / | / |
|  | 3-6 years | 25/71 (35.2) | 11/26 (42.3) | 2/7 (28.6) | / |
|  | Improvement | 25/25 (100.0) | 10/11 (90.9) | 2/2 (100.0) | / |
|  | Ineffective | / | 1/11 (9.1) | / | / |
|  | 7-10 years | 29/71 (40.8) | 10/26 (38.5) | 4/7 (57.1) | / |
|  | Improvement | 29/29 (100.0) | 10/10 (100.0) | 4/4 (100.0) | / |
|  | Ineffective | / | / | / | / |
|  | 11-15 years | 8/71 (11.3) | 2/26 (7.7) | / | 1/1 (100.0) |
|  | Improvement | 8/8 (100.0) | 2/2 (100.0) | / | 1/1 (100.0) |
|  | Ineffective | / | / | / | / |

Dual-use refers to patients who were initially treated with cefoperazone–sulbactam and subsequently switched to meropenem.
